# The Caspase 1 Inhibitor VX-765 Protects the Isolated Rat Heart via the RISK Pathway

**DOI:** 10.1007/s10557-018-6781-2

**Published:** 2018-03-26

**Authors:** Helison Do Carmo, Sapna Arjun, Orlando Petrucci, Derek M Yellon, Sean M Davidson

**Affiliations:** 1 Laboratory of Myocardial Ischemia/Reperfusion, Faculty of Medical Science, State University of Campinas–UNICAMP, Campinas, São Paulo Brazil; 2 The Hatter Cardiovascular Institute, University College London, 67 Chenies Mews, London, WC1E 6HX UK

**Keywords:** Caspase, Infarction, Ischaemic, Reperfusion, Kinases

## Abstract

**Purpose:**

Protecting the heart from ischaemia-reperfusion (IR) injury is a major goal in patients presenting with an acute myocardial infarction. Pyroptosis is a novel form of cell death in which caspase 1 is activated and cleaves interleukin 1β. VX-785 is a highly selective, prodrug caspase 1 inhibitor that is also clinically available. It has been shown to be protective against acute IR in vivo rat model, and therefore might be a promising possibility for future cardioprotective therapy. However, it is not known whether protection by VX-765 involves the reperfusion injury salvage kinase (RISK) pathway. We therefore investigated whether VX-765 protects the isolated, perfused rat heart via the PI3K/Akt pathway and whether protection was additive with ischaemic preconditioning (IPC).

**Methods:**

Langendorff-perfused rat hearts were subject to ischaemia and reperfusion injury in the presence of 30 μM VX-765, with precedent IPC, or the combination of VX-765 and IPC.

**Results:**

VX-765 reduced infarct size (28 vs 48% control; *P* < 0.05) to a similar extent as IPC (30%; *P* < 0.05). The PI3 kinase inhibitor, wortmannin, abolished the protective effect of VX-765. Importantly in the model used, we were unable to show additive protection with VX-765 + IPC.

**Conclusions:**

The caspase 1 inhibitor, VX-765, was able to reduce myocardial infarction in a model of IR injury. However, the addition of IPC did not demonstrate any further protection.

## Introduction

Protecting the heart from ischaemia-reperfusion injury is a major goal in patients presenting with an acute myocardial infarction. To date, it has been shown experimentally that protection against ischaemia-reperfusion (IR) injury can be mitigated by activating the reperfusion injury salvage kinase (RISK) pathway. However, in order to maximise such protection, it is important to identify non-RISK pathways that could be additive [1, 2].

Pyroptosis is a novel form of cell death in which caspase 1 is activated and cleaves interleukin 1β. The resulting IL1-β and IL-18 protein fragments are released from the cell via gasdermin D cell membrane pores [3]. In addition to stimulating an immune response, the released cytokines may potentially have a multiplying effect in stimulating death of local cardiomyocytes. Importantly, after myocardial IR, caspase 1 can be activated in both cardiomyocytes and cardiac fibroblasts, the major cell populations of the cardiac muscle [4, 5]. However, whether pyroptosis contributes to ischaemia-reperfusion injury has not been definitively established. The highly selective prodrug caspase 1 inhibitor, VX-765, has been shown to be protective against acute IR in vivo rat model [6]. This specific class of caspase inhibitor might be a promising possibility for future cardioprotective therapy [3]. However, it is not known whether VX-765 requires activation of known pro-survival kinases such as the phosphatidylinositol-3-kinase (PI3K)-Akt, one of the most prominent pro-survival signalling pathways associated with the RISK pathway which is known to protect against the damage caused by acute IR [7–9]. Interestingly, a well-known example of this is the phenomenon of ischaemic preconditioning (IPC) [10].

Therefore, in this study, we assessed the protective effect of the caspase 1 inhibitor, VX-765, in the presence of a PI3 kinase inhibitor. In addition, because of its well-defined mechanism of cardioprotection via the RISK pathway, we administered IPC simultaneously with VX-765 to ascertain whether this would result in additional protection against acute IR. The aim of this study was to determine whether caspase 1 inhibition could function via a different or complementary pathway to that of PI3K-Akt activation.

## Method

Male Sprague Dawley rats (300–350 g) were used according to UK (Scientific Procedures) Act of 1986. All animals received sodium pentobarbital (60 mg/kg) anaesthesia and a single dose of sodium heparin (0.1 UI). Following anaesthesia, the hearts were excised and retrogradely perfused with Krebs Henseleit buffer (mM: 118, NaCl; 25, NaHCO_3_; 11, D-glucose; 4.7, KCl; 1.22 MgSO_4_·7H_2_O; 1.21, KH_2_PO_4_; 1.84 CaCl2·2H_2_O) bubbled with carbogen (95 O_2_ and 5% CO_2_) at 37 °C and pH 7.4. The protocol applied was 40-min stabilisation followed by 35-min regional ischaemia with left anterior descending coronary artery occlusion and 2 h of reperfusion. The hearts were assigned to one of the following seven groups: (1) control-vehicle dimethyl sulfoxide (the solvent for VX-765); (2) IPC, consisting of 3 × 5-min cycles of ischaemia and reperfusion; (3) VX-765, (30 μM) given after 10-min stabilisation and throughout the ischaemia and reperfusion period; (4) control+W, control-vehicle group given wortmannin (100 nM) for 15 min at reperfusion; (5) IPC+W, IPC group followed by 15 min of wortmannin (100 nM) at reperfusion; (6) VX-765+W, VX-765 group followed by 15 min of wortmannin (100 nM) at reperfusion; (7) VX-765+IPC group, both VX-765 and IPC treatments given together. At the conclusion of the experiment, the analysis of infarct size (IS), as a proportion of area at risk (AAR), was calculated via planimetry using image J software (version 1.45, National Institutes of Health, USA). Infarct size was calculated a percentage of the area-at-risk (IS/AAR). Results were compared by nonparametric Kruskal–Wallis test (one-way ANOVA) with Dunn’s multiple comparisons post hoc test.

## Results

**Fig. 1:**
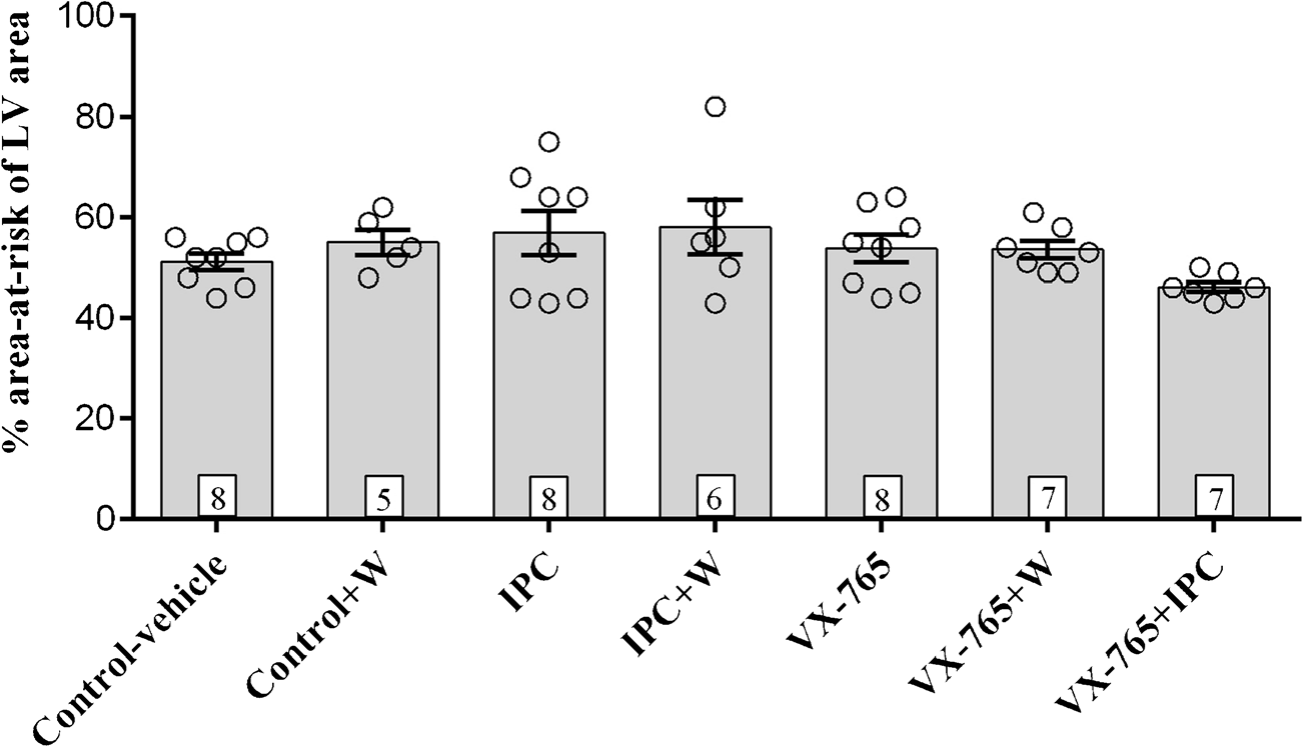
Area-at-risk as percentage of LV area, represented as scatter plot with bar graphs with a mean and standard error of the mean (SEM), and number of hearts in each group as indicated

**Fig. 2:**
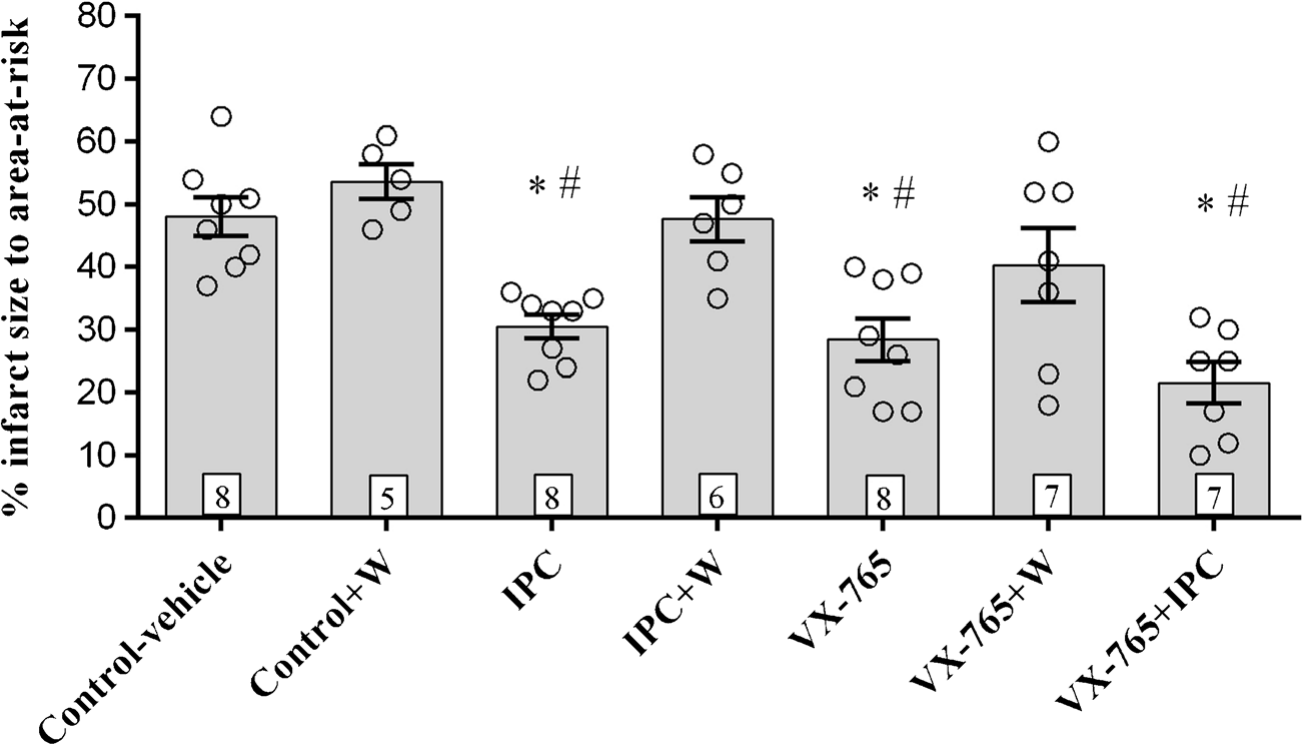
Infarct size as a percentage of area-at-risk represented as scatter plot and mean + standard error of the mean (SEM), with the number of hearts in each group indicated. The data was analysed by nonparametric Kruskal–Wallis test (one-way ANOVA) with Dunn’s multiple comparisons post hoc test. *P < 0.05 IPC, VX-765 and VX-765 vs control-vehicle; ^#^*P* < 0.05 IPC, VX-765 and VX-765 vs control + W

A total of 52 animals were used of which 3 were excluded due to lack of perfusion at the beginning of the protocol. The ischaemic area at risk was the same in all groups (Fig. 1). VX-765 reduced infarct size (28 vs 48% control-vehicle; *P* < 0.05) to a similar extent as IPC (30%; *P* < 0.05 compared to the control-vehicle group) (Fig. 2). Wortmannin, a non-specific PI3 kinase inhibitor, abolished the protective effect of VX-765 when given for 15 min at reperfusion (Fig. 2). Importantly in the model used, we were unable to show an additive protective effect with VX-765 + IPC (Fig. 2).

## Discussion

The present study assessed whether the caspase-1 inhibitor VX-765 could protect isolated hearts undergoing acute IR injury. The aim was to ascertain whether such inhibition is associated with the activation of PI3K-Akt and hence a conditioning-like mechanism or alternatively if it functioned via a mechanism independent of PI3K-Akt.

When activated, the majority of caspases result in apoptotic cell death. Importantly, caspase 1 has also been implicated in pyroptosis. IPC, as a trigger for pro-survival kinases, is associated with protection against necrosis, although there is also some evidence that it can inhibit the activation of other cell death pathways such as apoptosis [11]. In order to maximise the degree of protection in the setting of acute myocardial infarction, it is necessary to establish whether additional pharmacological approaches are able to elicit protection independent of the known phenomenon of IPC. Therefore, the potential for co-treatment with the caspase 1 inhibitor VX-765 and IPC was studied; such an approach was recently proposed and shown by Yang XM et al. using VX-765 plus the antiplatelet inhibitor Cangrelor [6].

However, in our study we showed that the protective effect of the prodrug, VX-765, was abolished by the PI3 kinase inhibitor, wortmannin. This demonstrates that this caspase 1 inhibitor appears to involve the same mechanism of protection as IPC: i.e. the PI3 kinase pathway. Furthermore, we were unable to demonstrate any additive protection when both treatments were administered together, suggesting that they both protect the heart via the same mechanism. The mechanism by which VX-765 activates PI3 kinase is not known, but may be either a consequence of caspase 1 inhibition or an off-target effect of the drug.

Infarct size has been assumed as a relevant endpoint in studies assessing the protective effect of agents in the setting of ischaemia-reperfusion injury. However, an experimental design using a single IR protocol may not fully predict the outcome. The present study used a protocol of 35-min regional ischaemia followed by 2-h reperfusion, which resulted in ~48% infarct size in control-vehicle group and an infarct size reduction to ~30% for both IPC and 28% for VX-765. It could be argued that in order to reveal any further reduction in infarct size, an extended ischaemic time (e.g. 60 min) may be necessary. In this regard, the translational value of the results remains to be demonstrated. In addition, it must be appreciated that Western blot analysis was not performed to confirm activation of the RISK pathway.

To summarise, we were able to demonstrate that the caspase 1 inhibitor, VX-765, was able to reduce myocardial infarction in a model of ischaemia-reperfusion injury. However, the addition of IPC did not demonstrate any additional protection.
